# Should Autism Spectrum Conditions Be Characterised in a More Positive Way in Our Modern World?

**DOI:** 10.3390/medicina56050233

**Published:** 2020-05-13

**Authors:** Barry Wright, Penny Spikins, Hannah Pearson

**Affiliations:** 1 Department of Health Sciences, University of York, York YO10 5DD, UK; 2 Hull York Medical School, University of York, York YO10 5DD, UK; 3 Archaeology PalaeoHub, Department of Archaeology, University of York, York YO10 5DD, UK; penny.spikins@york.ac.uk; 4 Child Oriented Mental Health Intervention Centre (COMIC), Leeds and York Partnership NHS Foundation Trust, York YO10 5NP, UK; h.pearson@nhs.net

**Keywords:** autism, autism spectrum condition (ASC), positive, diagnostic criteria, strengths, COVID-19

## Abstract

In a special issue that focuses on complex presentations related to Autism, we ask the question in this editorial whether an Autism Spectrum Condition without complexity is a disorder, or whether it represents human diversity? Much research into Autism Spectrum Conditions (ASCs) over the years has focused on comparisons between neuro-typical people and people with Autism Spectrum Conditions. These comparisons have tended to draw attention to ‘deficits’ in cognitive abilities and descriptions of behaviours that are characterised as unwanted. Not surprisingly, this is reflected in the classification systems from the World Health Organisation and the American Psychiatric Association. Public opinion about ASC may be influenced by presentations in the media of those with ASC who also have intellectual disability. Given that diagnostic systems are intended to help us better understand conditions in order to seek improved outcomes, we propose a more constructive approach to descriptions that uses more positive language, and balances descriptions of deficits with research finding of strengths and differences. We propose that this will be more helpful to individuals on the Autism Spectrum, both in terms of individual self-view, but also in terms of how society views Autism Spectrum Conditions more positively. Commentary has also been made on guidance that has been adjusted for people with ASC in relation to the current COVID-19 pandemic.

## 1. Background

Many papers are published about autism with comorbidities such as intellectual disability or other complex presentations. The literature is dominated by an approach where a very heterogeneous condition is presented as one overarching entity [1], perhaps leading to a perception that complexity is usually present in people with autism spectrum conditions (ASCs). This paper seeks to shine some light in this area.

Similarly, current diagnostic criteria see people with ASC through a particular lens that comes from and perpetuates a particular narrative about people with ASC. We explore here whether there is evidence for an alternative lens to be used.

## 2. What Is the Current Prevailing View of ASC?

### 2.1. Diagnostic Criteria

Autism spectrum conditions have been traditionally characterised in behavioural terms [2,3], including a series of impairments affecting social and emotional behaviours (such as difficulties in engaging in reciprocal conversation or in working cooperatively), communication (such as a literal understanding of language), imagination and flexibility (for example delays in empathy skills) and restricted repetitive patterns of behaviour and intense preoccupations. A primary focus on restrictions of behaviour and the interplay between culture and cognitive development, however, depends on cultural ideas of ‘normal’ [4]. Cultural expectations around attention, concentration and eye contact, for example, can place very different attributions on deviations from those expectations. Autism has been described as a disability which can severely impact upon quality of life [5]. People with autism have anxieties relating to change [6], and their parents often need additional support in their care and upbringing [7].

In the latest DSM-5 American Medical Association criteria [2], autism spectrum conditions are characterised more by the things that people with ASC cannot do, rather than by differences and strengths (shown in Table 1, reproduced from Spikins & Wright [8]). Diagnostic criteria focusing on negative characteristics may be associated with negative stereotypes being made about individuals with a particular diagnosis [9].

### 2.2. View of the General Public

A focus on psychologically defined deficits is very different from the understanding of difference found in studies of the general public. The United Kingdom (UK) general public appear to be fairly well aware of ASC, with Dillenburger et al. [10] finding an awareness rate of 82% when surveying adults living in Northern Ireland. Among respondents to the survey, females, those who had completed higher education and people with access to the internet were significantly more likely to be aware of ASC, whereas people from an ethnic minority background and those aged under 24 or over 65 were significantly less likely to be aware of ASC. Dillenburger et al. [10] found that many people could accurately report strengths of ASC, including intelligence, focus, determination and memory, as well as challenges including communication, social skills and social interaction. This suggests that adults in the UK are able to recognise positives of the condition. However, it is worth noting that one third of respondents did not comment on strengths of ASC, compared with only 13% who didn’t comment on challenges. There were also misconceptions around associating ASC with intellectual disability, with 30% of people responding definitely and 50% probably, when asked if ASC was a learning disability.

Awareness of ASC is also reasonably high among children and teenagers in the UK [11]. Children generally report positive attitudes to peers with ASC, and many identify bullying as an issue commonly faced.

### 2.3. Media Representation

There have been particular stereotypes presented in the media, such as in the film Rain Man, in the past, however the media representation continues to show a number of important biases. The media can have a significant impact on people’s opinions in the general population [12]. Research into media representations of ASC has found it often to be nonfactual and biased [13,14,15]. Common stereotypes portrayed of people with ASC include them being dangerous, out of control and unloved [15,16].

Huws and Jones [14] analysed the representation of ASC in British newspapers between 1999 and 2008; they found three main themes. Firstly, most accounts of ASC were from a third person perspective, and not from the individual with ASC themself. Most articles also focused on ASC as a childhood condition and cases of ASC in adults were rarely included. Secondly, ASC was generally represented in a negative way, and described using language such as ‘suffering’ and ‘victim’. Finally, where ASC was described more positively, there was regular mention of extraordinary and even ‘superhuman’ abilities, such as being able to memorise an extremely large amount of information and being able to calculate very advanced mathematical calculations mentally. Draaisma [13] suggests that this media portrayal of all/many people with ASC having a ‘super-skill’ may create the impression that those without are inadequate or do not have any positive features of autism.

### 2.4. Cultural Differences

Cultural differences play a significant role in understanding. Awareness of ASC appears to be high in other Western countries, such as France and the United States of America [17,18]. This seems to be at least partially due to national and global awareness campaigns such as Autism Awareness Month [17]. However, there are misconceptions present among these populations, for example over 20% of respondents to a questionnaire in France said that they believed parent-child interactions to be a risk factor for ASC [18].

Understanding of ASC appears to be more limited in some other countries. Many misconceptions around the understanding of ASC among teachers have been found in Oman, where the majority of participants believed that ASC is precipitated by maltreatment or neglect, and over 10% thought that “the majority of cases suffer from mental retardation” [19] (p. 4).

Rahbar et al. [20] investigated awareness of ASC among general practitioners (GPs) in Karachi, Pakistan, where only 46% had heard of ASC. Over 30% of GPs contacted in this study agreed with the statement: ‘Autism is a precursor for schizophrenia’. The authors suggest that this misconception came about due to earlier descriptions of ASC made by the American Psychiatric Association (APA) in the mid-20th century, implicating the impact that diagnostic criteria can have on public and medical perceptions going forward for many years.

Despite high awareness of ASC among the general UK population, this may not be the case for all subgroups. Dillenburger et al. [10] found that people from ethnic minorities were twelve times less likely to have heard of ASC, compared to those not from an ethnic minority. An example of cultural differences in attitude towards ASC within the UK can be found in the British-Somali community [21]. Qualitative research conducted with Somali migrants living in the UK has found that there is little acceptance of ASC as a condition among this population, and there is no word for ‘autism’ in the Somali language [21].

### 2.5. How Do People with ASC View ASC?

Many people with Asperger syndrome perceive themselves as different but not necessarily disabled [22], and are more likely than people without ASC to view autism as a positive or neutral neural difference [23]. When comparing the views of people with and without ASC, Gillespie-Lynch et al. [23] found that people with ASC described finding a cure as less important, and that many people with ASC did not view autism as a disease or disorder. Interestingly, the researchers also found that among all participants (both with and without ASC), an interest in curing autism was positively correlated with increased stigma towards people with the condition [23]. People with ASC tend to be more familiar with the DSM-V diagnostic criteria for ‘Autism Spectrum Disorders’ [2] than people without ASC [23]. Compared with people without ASC, people with ASC also more strongly disagree with the view that autism can be outgrown, and that people with autism are more violent than those without. People with ASC are also more likely to agree with the view that people with autism have empathy [23].

## 3. Is There Evidence to Challenge a Negative View of ASC?

### 3.1. Strengths and Skills of People with ASC

The teenage climate change activist Greta Thunberg, who has risen to fame in recent years by engaging school children in the climate change debate, describes her autism as a gift [24]. She credits some of her success to autism, describing her logical problem-solving abilities as a way of seeing a clear solution to climate change. She also describes her lack of interest in socialising as an explanation of her alternative, yet successful, tactics for gaining support for the movement [24]. Other popular public figures, such as Chris Packham, have also attributed their success to their autism [25].

People with autism show a range of talents and abilities. Many people with ASCs have a range of skills [26,27,28], such as memory, sensory perception or musical skills [26] or an ability to understand complex patterns or systems [29]. Some individuals with Autism Spectrum Conditions are reported to be more likely to have enhanced skills in areas such as mathematics [30], music [31], visual perception [32,33], heightened touch [29], increased auditory capacity [34] or olfactory sensitivity [35].

Some individuals with autism are good at spotting details in their sensory environment, as seen, for example, in embedded figures tests or an ability to find detail in environmental patterns [36,37,38]. These strengths may make people with ASC particularly suited to working in fields such as mathematics, information technology and natural sciences. The proportion of people with ASC studying and working in these fields is already greater than that of neuro-typical populations [39].

So what of the differences in empathy skill development, (so called ‘deficits’ in empathy or theory of mind [40]). Could it be that these may be advantageous in some situations? In a crisis for example some people with autism may be logical [7] and less influenced by high levels of emotionality that may impair coping. People with ASC may be more drawn to fairness and justice [41] with evidence that individuals with Asperger syndrome or High Functioning Autism are more likely to be drawn to the legal profession [7]. With less need to seek approval or please others [42] it may be easier to pursue justice without prejudice, or be less likely to back down from the truth of an argument when experiencing group pressure or disapproval [43].

It is also important to remember that in many areas people with ASC have no advantages or deficits compared to neuro-typical people and perform in very similar ways. For example, Kirchner et al. [44] analysed character strengths, using the Values in Action Inventory [45], in individuals with and without a diagnosis of autism. They found a few significant differences, with people with ASC more likely to have creativity as a key strength, and with more common strengths in those without ASC described as love (defined as “valuing close relationships with others” [46] (Table 1)) and humour. However, they found no significant differences in the presence of twenty-one other character strengths [44].

Similarly, Lorenz and Heinitz [39] examined occupational strengths in adults with and without ASC, finding a significant increase in prevalence of ten strengths for those with autism including: attention to detail, logical reasoning, focus, systemising, consistency, visual skills, retentiveness, repetitive tasks, numbers and auditory skills. Six strengths were found for those without ASC (verbal skills, flexibility, social skills, multitasking, empathy and team work), but there were no significant differences in ten other areas that included: reliability, creative solutions, organising ability, apprehension, stamina, pro-activeness, fine motor skills, conscientiousness, emotional control and physical work. This shows that in many areas people with ASC are not disadvantaged compared to neuro-typical people, with each bringing complimentary skills to the community.

### 3.2. How Long Has ASC Been Around in History/Prehistory?

There is evidence that Autism Spectrum Conditions have been present in human society for thousands of years [8,47]. Given that evolution can be brutal at removing unwanted genes [48,49,50] this suggests that some traits of autism may carry evolutionary advantages [51,52]. Autism spectrum conditions (ASCs) are likely to be part of natural human variation [53,54,55,56,57]. It has been argued that if autism was a ‘disorder’ it could not have been supported in past societies [58].

Happé and Frith [59] argue that given the advantages that an extreme cognitive focus on detail can generate, the persistence of such individuals within the gene pool “is not hard to explain” (p. 16) a point argued extensively in the book The Prehistory of Autism, by authors Spikins & Wright [8]. It is proposed that technological skills and understanding might have given advantages to hunter gatherer groups, as they do for modern day advances in computer science. Knowledge or memory of important information to do with food resources and their availability or weather changes bring significant advantages to groups, especially at times of hardship or limited resource availability [60].

### 3.3. Is There a Bias in the Way We Have Conceptualised ASC?

#### 3.3.1. Do We Conflate ASC and Intellectual Disability?

In the 1970s, the mean IQ of individuals reported as diagnosed with autism was approximately 60 [61]. In retrospect, we now know that these were individuals with autism who had a co-morbidity of intellectual disability, since individuals with ASCs can have an IQ across the full possible range. Indeed ASC associated with intellectual disability is not the most typical form of the condition [62,63]. The more common form of ASC is not associated with intellectual disability [62,63]. ASC without intellectual disability (often referred to as Asperger Syndrome or High Functioning Autism and increasingly Autism Spectrum Disorder (mild)) does not necessarily require care or support [22] and is highly heritable [64]. Autism spectrum conditions (ASCs) occur in approximately 1.6% of the population [65,66].

#### 3.3.2. Is Autism Really Asocial?

Diagnostic systems usually characterise a central ‘deficit’ of ASC as being large difficulties in social reciprocity, and refer to ASCs as having many social deficits including “deficits in developing, maintaining and understanding relationships and an absence of interest in peers” [2] (299.00 (F84.0)). Recent research suggests that this early clarification is not accurate. It is likely to be based on complex presentations of ASC, including those with intellectual disabilities.

More recent research suggests that the characterisation of ASC as an asocial condition is not accurate. Most adults on the autism spectrum without intellectual disability are more socially integrated than previously estimated. Individuals with ASC are known to follow social rules, analytically rather than intuitively [67]. They can also have healthy roles and function in society [68], for example in spheres such as engineering, mathematics, physics, information technology and law [7,69,70]. Individuals with Asperger syndrome often form partnerships and have children [64,71], in common with their neuro-typical counterparts.

Recent research suggests people with ASC are not asocial but ‘differently’ social [72,73], in that they tend to be selective in the way that they socialise [74]. They often prefer different styles of social engagement, basing them around common specific interests or around discussions of factual or analytical perspectives or interests with colleagues, friends or family. Whilst people with ASC may shy away from face-to-face narratives or emotional displays [51,70], they may enjoy factual informational exchange and be drawn to communicating through technology [72,73].

How we measure quality of life [55], and ‘social’ expectations of behaviour also influence the perceptions around autism. For example, some of the questions asked in quality of life measures (so often held up as benchmarks) explore participation in organised activities. People with ASC may avoid organised activities in large groups of people, but may nonetheless be very productive and happy. They may prefer 1:1 interaction around a hobby or an interest. There are cultural and neuro-typically defined assumptions about what good quality of life looks like.

## 4. Could We Describe ASC in a Better Way?

### 4.1. Difference or Disability?

Neuroimaging studies suggest differences in cortical connectivity [75,76,77,78,79]. These differences are important in sensory perception and interpretation of our sensory world [80]. For example, individuals with autism share a focus on detail [59]. Individuals with autism also perceive the natural and social world in analytical terms, as systems with rules and patterns to analyse, rather than relying on empathising or intuitive means of interpretation [81,82]. These differences in skills between the analytical mind and the strongly empathising mind that have been described [51,81] should not imply one to be superior to the other, since both skills are important in a community.

**Figure 1:**
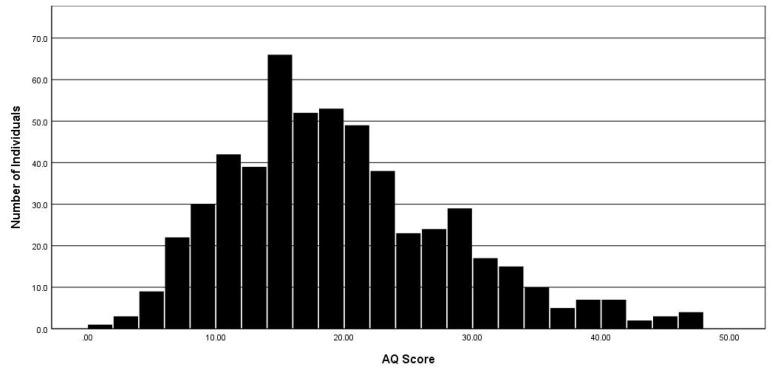
AQ scores of 557 undergraduate students at an English University who participated in an archaeology survey.

We recently sampled 557 university students in the North of England and asked them to complete an Asperger Questionnaire [83], and this interestingly showed a continuous variation (see Figure 1, credit: Callum Scott, based on data presented in Spikins et al. [84]), which is in line with another very similar university study [83] and other population studies (n = 6000) [85]. These studies and studies like them appear to confirm that autism symptomatology is common across society and woven into it [86,87].

### 4.2. How Do We Represent All This in Diagnostic Systems?

Given the discussion above should the definitions of ASCs be amended to include some of the positive aspects of ASCs to create a more balanced picture? For example, they might look like this (see Table 2, reproduced from Spikins & Wright [8]):

## 5. 2020. COVID-19 Pandemic

### 5.1. Guidance Related to the 2020 COVID-19 Pandemic

There has been significant disruption to everyday life as efforts to minimise the effects of the 2020 COVID-19 pandemic have been introduced across the UK [88] and many other nations of the world. Many of the measures introduced are likely to have a detrimental effect on the mental health and wellbeing of people with ASC. A need for structure and routine is a key part of the DSM-V diagnostic criteria for ASC [2]; a need which is likely to be significantly affected by social isolation and school and workplace closures. People with ASC are also more likely than neuro-typical people to suffer from anxiety [89,90].

Initial Covid-19 guidance from the National Institute for Health and Care Excellence (NICE) [91] advised the use of the Clinical Frailty Scale (CFS) [92] to assess frailty (to determine appropriateness of critical care versus end of life care) for people aged over 65. NICE specifically recommended that an individualised assessment of frailty be used for anyone aged over 65 “with a long-term physical disability, learning disability *or autism*” [91]. A CFS score is used to consider whether or not certain choices at end of life care are appropriate [91], with an individual scoring 5 or more described as needing help with higher order cognitive tasks, such as managing finances and some physical tasks, such as heavy housework [92]. It is unclear why people with autism need to be separately considered under these guidelines. Although many people with autism may score highly on the CFS, this is likely to be due to a comorbid condition, such as physical or intellectual disability, which are already covered in the guidance. As the diagnostic criteria for autism only includes difficulties in social functions and restricted/repetitive patterns of behaviour [2], is it appropriate for people with autism without intellectual disability or any other comorbid conditions to be considered separately from neuro-typical individuals? Could it be that the inclusion of autism in these guidelines is due to the widespread misconception that autism is *always* associated with intellectual disability and/or a lack of independence? It is likely that the analytical skills of research colleagues with ASC may be very helpful in planning the response to COVID-19 or in researching scientifically based treatments for it.

Other COVID-19 related guidance has been adjusted for people with ASC. The UK government currently advise that individuals should only go outside for exercise once a day and that they should not travel far from their home to do this [93]. People with a significant health need, described as including a learning disability *or autism*, are exempt from this directive. This guidance is more appropriate as it is addressing a genuine need related to ASC, the need for structure and routine [2]. Some people with ASC are also more sensitive to sensory stimuli [29] and travel may be required to find an appropriately quiet place to exercise. Likewise, the allowance of a visitor in hospital (when visitors in most situations have been suspended) by NHS England [94], is also appropriate, as it addresses the fact that many people with ASC are less stressed when a familiar person is present [95].

### 5.2. How Do People with ASC Respond in a Crisis?

As with the neuro-typical population, people with ASC may respond to the COVID-19 pandemic in different ways. Many people with ASC do not respond well to change, and enjoy consistency in daily routines [96,97]. The current limitations on daily life, such as school and workplace closures, are likely to disrupt these routines, and have a negative impact on the mental health of many people with ASC. The way that people socialise has also had to change, with most interaction with individuals outside a person’s household now taking place virtually. For some people with ASC, this may be a welcome respite from the difficulties faced when socialising [97], and for many this could actually provide a benefit, whereby socialising is now taking place in a format that they are more comfortable with [72,73]. 

As we consider the negatives and challenges, we should also ask the question whether there any ways in which ASC confers advantages when dealing with a crisis? An ability to think logically [7], understand complex systems [29] and be analytical [39] may helpful as COVID-19 responses are considered. Motivation to follow rules and regulations [98] may be protective in the context of government guidelines.

## 6. Summary

Should some consideration be given to how we conceptualise and describe ASC that takes into account difference, strengths and the perspectives of the people we are giving diagnoses to? Would this help in developing support systems that enable people with ASC within society, rather than the impossible task of trying to change them into neuro-typical people? We recommend a new positive approach to ASC that recognises difference and moves away from conflating people with ASC *with complex needs or intellectual disability* with those who have ASC *without intellectual disability* and enables families, clinicians, researchers and people with ASC themselves to consider more constructive approaches going forward.

## Figures and Tables

**Table 1:** Extracts from American Psychiatric Association [2] Criteria for Autism Spectrum Conditions.

Persistent Deficits in Social Communication and Social Interaction Across Multiple Contexts, As Manifested by:
**Deficits** in social-emotional reciprocity… **failure** of normal back-and-forth conversation… **failure** to initiate or respond to social interactions.
**Deficits** in nonverbal communicative behaviours… **poorly** integrated verbal and nonverbal communication… **abnormalities** in eye contact and body language or deficits in understanding and use of gestures… **lack** of facial expressions and nonverbal communication.
**Deficits** in developing, maintaining, and understand relationships… **absence** of interest in peers.
**Restricted**, repetitive patterns of behaviour, interests, or activities, as manifested by **inflexible adherence** to routines… rigid thinking patterns.
**Highly restricted**, **fixated** interests that are abnormal in intensity or focus
**Hyper or hypo-activity** to sensory input.

**Table 2:** An alternative set of criteria for autism spectrum disorder.

**Differences in Social Communication Compared with Neuro-Typical People Such As:**
Logical approach to appraisal of socio-emotional situations.
Utilitarian approach to the need for communication.
Preference for communicating only when it is necessary to achieve an outcome (often using written or electronic communication in preference to verbal and nonverbal communication).
Stronger reliance on environmental information than eye contact and body language.
Small close group of functional relationships in preference to larger group of social acquaintances
**Differences in Patterns of Interest and Occupation, as Manifested by**:
Liking for structure and routine.
A tendency to an interest in facts, details, categorisation, patterns, visual or topographical memory, numeracy and how things work.
Differences in interaction with the sensory environment including ability to perceive patterns and details that others can’t easily perceive.
A tendency to like rules and logic.
